# Genotype- and tissue-specific metabolic networks and hub genes involved in water-induced distinct sweet cherry fruit cracking phenotypes

**DOI:** 10.1016/j.csbj.2021.09.030

**Published:** 2021-09-28

**Authors:** Michail Michailidis, Evangelos Karagiannis, Christos Bazakos, Georgia Tanou, Ioannis Ganopoulos, Athanassios Molassiotis

**Affiliations:** a Laboratory of Pomology, Department of Horticulture, Aristotle University of Thessaloniki, Thessaloniki-Thermi 57001, Greece; b Institute of Plant Breeding and Genetic Resources, ELGO-DEMETER, Thessaloniki , Greece; c Joint Laboratory of Horticulture, ELGO-DEMETER, Thessaloniki-Thermi 57001, Greece; d Department of Comparative Development and Genetics, Max Planck Institute for Plant Breeding Research, Carl-von-Linne-Weg 10, Cologne 50829, Germany; e Institute of Soil and Water Resources, ELGO-DEMETER, Thessaloniki-Thermi 57001, Greece

**Keywords:** Abscisic acid, Ethylene, Expansins, Flesh and skin tissues, Fruit cracking, Metabolomic, Pectin, Sweet cherry, Transcriptomic, Water dipping

## Abstract

Sweet cherry fruit cracking is a complex physiological disorder that causes significant economic losses. Despite many years of research there is a lack of understanding of the mechanisms involved in cracking. Here, skin and flesh tissue from the cracking susceptible 'Early Bigi’ and the cracking tolerant ‘Regina’ cultivars were sampled prior and just after water dipping treatment to identify water-affected metabolic networks that putatively involved in fruit cracking. Primary metabolites, most strongly those involved in sugars and amino acid metabolism, such as glucose and asparagine, shifted in 'Early Bigi’ compared with ‘Regina’ tissues following water exposure. Comparisons between cultivars, tissues and dipping points identified significant differentially expressed genes. Particularly, genes related to abscisic acid, ethylene biosynthesis, pectin metabolism, expansins and aquaporins were altered in water-exposed tissues. To further characterize the role of these genes in cracking, their single nucleotide variants of the coding regions was studied in another eight sweet cherry cultivars, which differ in their sensitivity to cracking, revealing a strong link mainly between pectin metabolism-related genes and cracking-phenotypes. Integrated metabolomic and transcriptomic profiling uncovered genotypic- and tissue-specific metabolic pathways, including tricarboxylic acid cycle, cell enlargement, lipid and ethanol biosynthesis, and plant defense that putatively are involved in fruit cracking. Based on these results, a model which describes the skin and flesh metabolic reprogramming during water-induced fruit cracking in the susceptible 'Early Bigi’ cultivar is presented. Τhis study can help to explore novel candidate genes and metabolic pathways for cracking tolerance in sweet cherry.

## Graphical abstract

**Figure:**
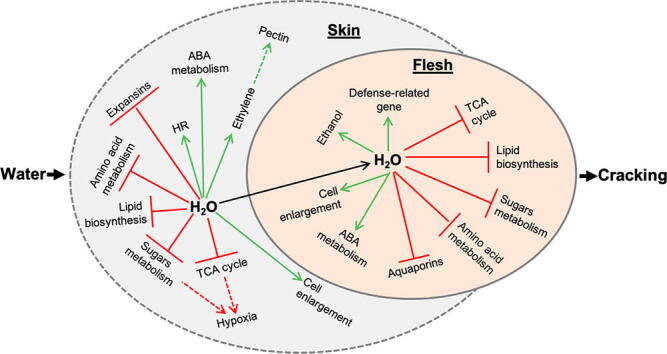

## 1 Introduction

The rain-induced cracking of sweet cherry fruit is a serious environmental problem in many regions of the world, where this high-value fruit crop is produced [1], [2], [3], [4]. A long-standing hypothesis holds that fruit cracking are caused by direct and probably localized water intake through fruit cuticle, resulting in the bursting of individual flesh cells and the consequent leakage of cell contents into the apoplast [5]. However, contradictory studies have generated several modified hypotheses. It has been recently proposed, for example, that sweet cherry cracking is primarily a function of wetness duration and the percentage of the wetted surface area [6].

The cracking of sweet cherry (*Prunus avium* L.: *Rosaceae*) fruit has challenged the scientific community for decades. The first systematic studies about sweet cherry cracking were carried out >90 years ago [7] and since then, a high number of studies with the fundamental aim to classify the sweet cherry cultivars based on their susceptibility to cracking have been following [3], [8], [9], [10], [11]. Three types of skin cracking have been reported in the literature, stem end cracks, top-end cracks, and common lateral cracks [8], [10], which is possibly dependent on different ways of water uptake in fruits [12]. In parallel, the exocarp (skin) properties of sweet cherries regarding water influx have been studied [13], [14], [15]. Specifically, the sweet cherry skin consists of a thin layer of the epidermis with at least eight layers of cells. The outer part of the skin (epidermis) includes on the external layer, hydrophobic substances, and an inner layer of hydrophilic substances, such as polyurines and glucans, with a single layer of cellulose. Stomata in fruits (85–200 cm^2^) are much less compared to the leaves (5000–10000 cm^2^), however, fruit epidermis contains pores that water molecules move through these [13], [14], [15].

Although it has been proposed that many genes might be involved in the process of fruit cracking [16], only a few sweet cherry genes, such as lipid transfer protein (*PaLTPG1*), wax synthase (*PaWS*) and 3-ketoacyl-CoA synthase (*PaKCS6*) have been demonstrated to be mainly associated with the biosynthesis of cuticular wax and possibly with cracking development [17], [18], [19]. Additionally, changes in the content of a few metabolites, such as fucose and taxifolin, could be correlated with the cracking susceptibility in various sweet cherry cultivars [8]. Previous studies have shown that plant hormones, like abscisic acid (ABA) and ethylene as well as genes encoding cell wall enzymes related to pectin metabolism and cell expansion (expansins) play an important role in cracking mechanism in various fruits [1], [17], [20], [21], [22], [23], [24]. However, considering the importance of water-induced cracking in sweet cherry fruit, the lack of molecular information on this biological phenomenon is surprising.

Similarly to other stone fruit, the edible, fleshy part of the sweet cherry fruit consists of exocarp (skin) and mesocarp (flesh). Flesh and skin are highly specialized tissues that both involved in water uptake during rain-induced cracking in sweet cherry fruit. These tissue types also exhibited clear evidence of functional and metabolic specialization, such as the presence of a thick hydrophobic cuticle coating the outer epidermis, spatially distinct cell expansion rate and osmotic potential [25], [26]. Such obvious morphological features hint at extensive and complex tissue-related variation in gene expression and possibly in water uptake and cracking behavior [19].

Fruit cracking is a highly complex phenomenon which has been related to many cultivar-related factors, including fruit firmness, skin characteristics, fruit water uptake and osmolarity [8]. Although it is well known that cultivars differ in their susceptibility to cracking, only limited information is available to explain this feature [8], [9], [10]. Tolerance to cracking is a complex quantitative trait that is possibly controlled by several genes and is often confounded by the phenotyping of cultivar differences in terms of cracking susceptibility/tolerance [11], [16], [19]. Therefore, an examination of how cultivars specifically differ in their cracking susceptibility might lead to a greater understanding of some of the fundamental processes involved in fruit cracking. Here, we aimed (i) to characterize how skin and flesh metabolism is reprogrammed by water uptake, and (ii) to identify differentially expressed genes and metabolic pathways that contribute to cracking sensitivity/tolerance following water uptake in sweet cherry fruit. To achieve this, we sampled different tissues (skin and flesh) of the cracking-susceptible (‘Early Bigi’) and the cracking-tolerant (‘Regina’) cultivars [8], prior and just after water dipping treatment (herein referred as ‘pre-water’ and ‘post-water’ dipping). Using single nucleotide variants analysis and cracking data of various cultivars we also provide genomic-based evidence that pectin metabolism-related genes involved in sweet cherry cracking. Based on putative functions of the differentially expressed genes and the identified metabolites, a potential genotype-specific water-induced cracking mechanism in the sweet cherry fruit is proposed.

## 2 Materials and methods

### 2.1 Fruit material, water treatment and sampling

**Fig. 1:**
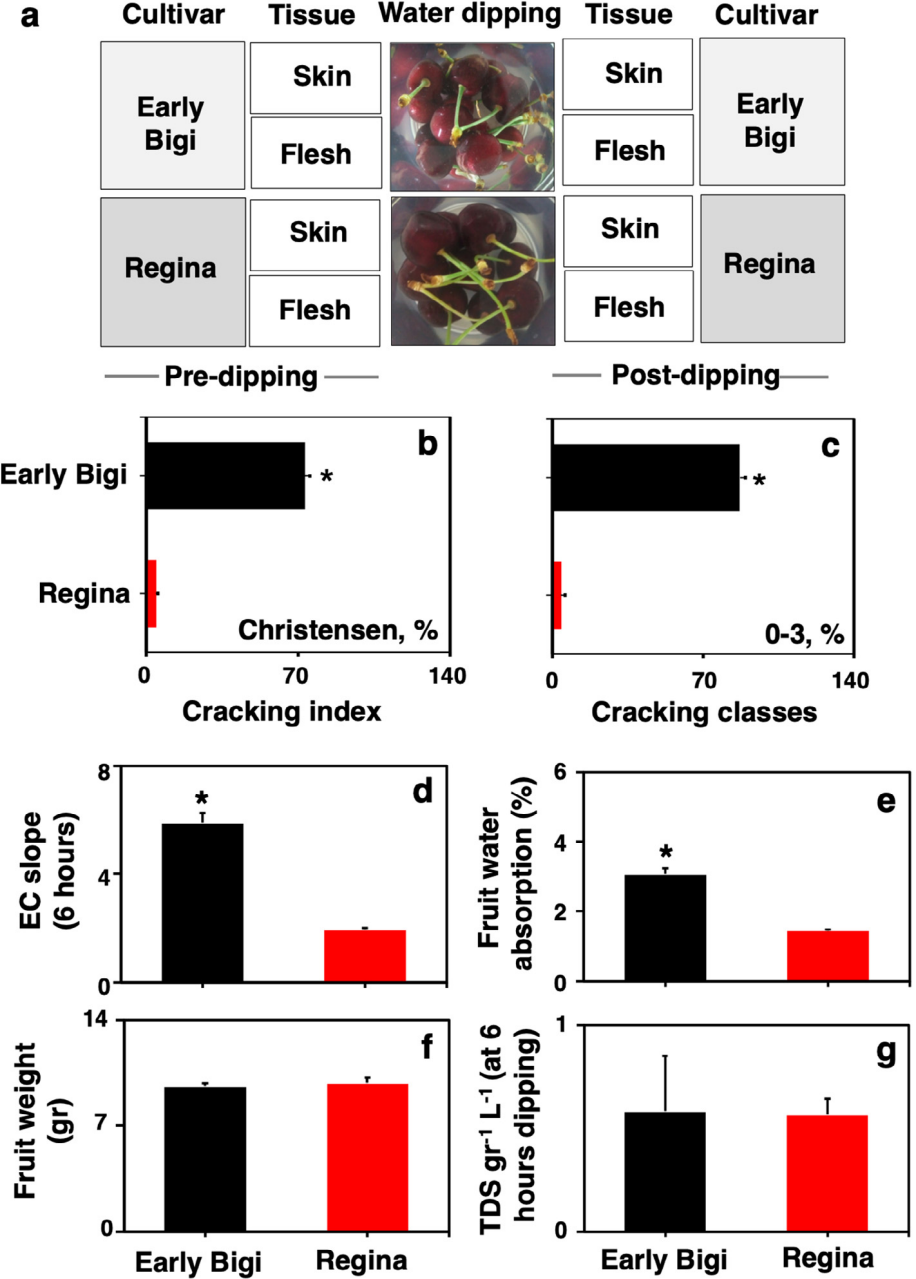
(a) A schematic presentation of the sampling process. Fruits of sweet cherry cultivars ‘Early Bigi’ and ‘Regina’ were exposed to water dipping for 1 h just after harvest. Skin and flesh tissues were sampled prior (pre-dipping; PreD) and immediately after water dipping (post-dipping; PostD). Cracking-related parameters of fruits exposed to water treatment; (b) cracking index via hourly observations, (c) cracking classes, (d) electrical conductivity (EC) slope during 6 h of dipping, (e) fruit water absorption, (f) fruit weight (g) total dissolved solids (TDS) in dH_2_O after 6 h of dipping. Each bar represents the mean of 3 biological replicates in a bunch of 20 fruits per replicate and lines indicate standard deviation (SD). Differences between two cultivars were detected based on t-Student test; *P ≤ 0.05.

Fruits of the two sweet cherry cultivars ‘Early Bigi’ and ‘Regina’ were harvested. The cv. Regina (cross of the 'Schneiders Spate Knorpelkirsche' and 'Rube') is a late-season cherry from Germany while ‘Early Bigi’ is a very early season French cultivar and its origin is an unknown varietal crossing. Details regarding their fruit characteristics are provided in our previous work [8]. Sweet cherry trees were ten years old trees grafted onto MaxMa 14 rootstock (trained in open vase) growing in a commercial orchard located at North Greece (Central Macedonia – Edessa) in 2017–2018 season. At commercial harvest stage (for ‘Early Bigi’ at 30.05.2018 and for ‘Regina’ at 02.07.2018; the fruit ripening characteristics are given in Supplementary Fig. 1), three hundred fruits with no visual defects of each cultivar were collected. Immediately after harvest, a batch of thirty fruits in triplicate per cultivars were exposed to water dipping treatment for one hour. Skin tissue was separated from flesh tissue with a razor blade prior and samples of both tissues (skin and flesh) were taken prior to water dipping (defined as ‘pre-dipping’; PreD) and immediately after this treatment (defined as ‘post-dipping’; PostD). Subsequently, samples were immersed in liquid nitrogen and stored at −80 °C for further analysis. A scheme summarizing the experimental system and the fruit sampling procedure is depicted in Fig. 1a.

### 2.2 Fruit ripening characteristics

Fruit skin color indicators, namely Lightness (L*), Chroma (C*) and Hue angle (H*), were measured with a Minolta CR200 colorimeter (Minolta, Osaka, Japan) using the CIE (Commission International de l’Eclairage) parameters were evaluated for both cultivars at harvest. Each measurement was conducted at two opposite surfaces of each fruit and the calculated means were presented. Total soluble solids (TSS) concentration was determined with electronic refractometer (Atago PAL1, Tokyo, Japan) while titratable acidity (TA, % malate) was determined by potentiometric titration with 0.1 N NaOH up to pH 8.2; Ripening index (RI) was calculated by the equation: TSS TA^−1^. For each analysis, three replicates of twenty fruits were determined (Fig. 1 & Supplementary Fig. 1), as previously described [27], [28].

### 2.3 Fruit cracking determination and water-uptake traits

Cracking was estimated by ‘Christensen method’ as described in detail [8] in five replicates of twenty fruits and the results were expressed as cracking index (%). In brief, fruits immersed in distilled water (at 20 °C) for a total period of 6 h with the hourly recording of skin cracking incidence, and then the percentage of cracking index was determined by the following equation: [$\sum_{i=1}^{6}\left(7-i)\;x\;\mathrm{cracked}\;\mathrm{fruits}\;\mathrm{in}\;\mathrm{hour}\right)$/6× total fruits] ×100. Thereafter, in the same five replicates of twenty fruits, cracking classes in sweet cherries skin were determined, after 6 h of dipping by the following equation: [$\sum_{i=1}^{a=total\;fruits}\left(i\;x\;class\;of\;fruits\right)$/3× $\sum_{i=1}^{a=total\;fruits}\left(i\right)$] ×100; with total fruits = 20; 0 class indicate absence of cracking; 1st class, cracking with area ≤ 3 mm^2^; 2nd class, cracking with area >3 mm^2^ and ≤ 6 mm^2^; 3rd class, cracking with area >6 mm^2^. Results were expressed as cracking classes (%).

In the same batch of fruits, fruit water absorption, electrical conductivity (EC), slope and total dissolved solids (TDS) were determined. For water absorption analysis, fruit tare before and after six hours of water immersion was determined, droplets were removed by centrifuge and airflow for 1 min and the results of fruit water absorption expressed as a percentage (%). Hourly measurement of water EC was conducted using an EC meter (HI 9033 and HI 76,302 Hanna Instruments Inc. USA) for 6 h during fruit immersion and constructing a linear model (6 EC points or less for each replicate) where the slope of the line calculated. Following 6 h water-immersion, TDS were determined as described [29] and expressed as TDS per gram of fruits (gr^−1^) per liter (L^−1^).

### 2.4 Primary metabolites profiling

Polar metabolites in each time point, tissue and cultivar were determined in three biological replicates, as previously described [30]. Frozen flesh or skin samples (500 mg) were extracted with 1.4 mL methanol plus 0.1 mL adonitol (1 mg mL^−1^) in 2-mL screw cap, thereafter extraction processing was carried as described in our previous study [31]. Gas chromatography–mass spectrometry (GC–MS) analysis process was performed using a PerkinElmer Clarus® 590 GC equipped with Clarus® SQ 8 S MS (USA) as described [32]. Injection of 1 μL sample with a split ratio of 20:1 was conducted on a TR-5MS capillary column 30 m ×0.25 mm ×0.25 μm (Supplementary Table 1). The peak area integration and chromatogram visualization were performed using TurboMass^TM^ processing program. Standard compounds or NIST11 and GOLM database [33] in case of unknown peaks were used for qualitative and quantitative determination of polar metabolites. Detected metabolites were expressed as relative abundance of the adonitol (internal standard), and the data were provided in the Supplementary Table 1.

### 2.5 Total RNA extraction, library construction and RNA-seq analysis

Total RNA from 3 biological replicates of both skin and flesh samples of the two sweet cherry cultivars at two timepoints was isolated by using the RNeasy® Plus Mini Kit from Qiagen (Valencia, CA, USA). TruSeq stranded libraries with polyA enrichment were prepared from the isolated mRNA following manufacturer’s instructions. The quality of isolated RNA and library was assessed with Agilent Bionanalyzer (RIN >7) and the libraries were sequenced on an Illumina® HiSeq 3000 platform (∼13.5 M Single-End reads 150 bp/sample).

The single-end reads were aligned to the reference genome (Prunus avium, GCF_002207925.1_PAV_r1.0_genomic) using Hisat2 with default parameters. Raw read counts per gene were quantified with HTSeq v0.6.1p1 (http://www-huber.embl.de/users/anders/HTSeq/) using the ‘-s reverse --type = gene’ option. The raw reads were normalized with the metric “Transcripts Per Million (TPM)”. The normalized data was filtered to leave only the always-above-0 genes and then it was log2 transformed and scaled (z-score: mean center and divide by standard deviation). The normalized data was used for the Principal Component Analysis (PCA) and Hierarchical Clustering (HC). PCA was performed with the function ‘prcomp’ in R (version 3.6.2). The Euclidean distance metric was used for HC. The samples and the genes were clustered with Spearman and Pearson correlation methods, respectively and the hierarchical cluster analysis was performed with the function “hclust” using the method “complete” in R (version 3.6.2). Differential expression between samples and cultivars was determined using the package edgeR [34], that implements exact statistical methods, giving as an input the raw data. Genes with q-value (p-value adjusted) below 0.01 were called significant differentially expressed genes without any threshold based on fold change.

### 2.6 qRT-PCR validation experiment

Extraction of mRNA was conducted in three biological replicates using the RNeasy® Plus Mini Kit from Qiagen (Valencia, CA, USA) [35], [36]. To obtain cDNA, 10 ng RNA was reverse transcribed using the Superscript^TM^ II reverse transcriptase kit (200 U, Life Technologies, Inc.) and a PCR (ProFlex; Thermo Fisher Scientific, Inc.). Real-time PCR was performed using 2 μL cDNA, 0.4 μL for forward and reverse primers and following the instruction of PowerUp^TM^ SYBR® Green Master Mix (Applied Biosystems, Austin, TX, USA) in a QuantStudio® 5 Real-Time PCR System (96-well, Thermo Fisher Scientific). Primers were designed with Primer3Plus (http://www.bioin forma tics.nl/cgi-bin/prime r3plu s/prime r3plu s.cgi) (Supplementary Table 5). The qPCR program was performed as previously described [35], [36]. Ct recorded at 0.15 ΔRn and melt curve to verify PCR products. Data were analyzed using ΔΔCt method [37] and were expressed as −ΔΔCt.

### 2.7 Single nucleotide polymorphism (SNP) calling and annotation

The genotyping data of the eight sweet cherry cultivars, namely Bakirtzeika (Bak), Mieza (Mie), Proimo Kolindrou (PrKld), Tragana Edessis (TrEd), Tragana Edessis Naousis (TrEdNa), Tragana Rodochoriou (TrRd), Tsolakeika (Tsol), Vasileiadi (Vas) was obtained from our previous study [38] while the cracking index of these cultivars was also evaluated (Supplementary Table 4). Briefly, SNP calling and annotation was performed based on the *P. avium* Genome v1.0.a1 (https://www.rosaceae.org/species/prunus_avium/genome_v1.0.a1) using snpEff tool [39]. GATK genome analysis toolkit (version 4.1.4.1; https://hub.docker.com/r/broadinstitute/gatk/) was used to call SNPs by using standard hard filtering parameters of HaplotypeCaller. For the purpose of the current study, the SNPs at the open reading frames (ORFs) were only studied. The SNPs in coding regions were further classified as synonymous, missense, splice site gain, stop codon gain and stop codon loss. The SNPs of selected hub genes were analyzed with GenAlEx6.503 [40]. Principal Coordinate Analysis (PCoA) was obtained from genetic distance measurements in GenAlEx. The phylogenetic tree was constructed using unweighted pair-group method with arithmetic mean (UPGMA) cluster analysis based on the Nei's genetic distance using MEGA X software [41].

### 2.8 Statistical analysis

One-way analysis of variance (ANOVA) conducted using SPSS (SPSS v21.0., Chicago, USA). Mean values of ripening traits and metabolites were compared by *t*-test (*P* ≤ 0.05) and gene expressions for validation were compared based on Duncan’s Multiple Range Test (*P* ≤ 0.05). Clustering correlation was conducted using ClustVis software [42]. Metabolites and transcripts were mapped to KEGG database in case to fold changes (metabolites; log_2_FC < or >0, transcripts; log_2_FC > |2|) or significance (metabolites; *p-value* ≤ 0.05*,* transcripts; *q- value* ≤ 0.05).

## 3 Results

### 3.1 The cracking characteristics of the 'early bigi' and 'regina' are discrepant

Following commercial harvest (ripening and color indexes of both cultivars are provided in Supplementary Fig. 1), the cracking behavior of the two sweet cherry cultivars, namely ‘Early Bigi’ and ‘Regina’, were evaluated by the immersion of their fruit in water for one hour. Both cracking index and cracking classes (Fig. 1b and c) were higher in ‘Early Bigi’ compared to ‘Regina’. It is noted that ‘Early Bigi’ fruits exposed to water displayed cracking symptoms (Supplementary Fig. 1); this cultivar therefore is considered to show ‘cracking-affected phenotypes’. By contrast, ‘Regina’ fruits treated with water did not show visible cracking damage; this cultivar is herein defined as showing ‘cracking-unaffected phenotypes’. Although weight and overall TDS remained unchanged by water immersion (Fig. 1f and g), the fruit water absorption and EC-release slope on the water were significantly increased in ‘Early Bigi’ fruit exposed to water treatment (Fig. 1d and e), further supporting that these cultivars show distinct water-responsive cracking features under the current experimental conditions.

### 3.2 Fruit primary metabolism in response to watering episode

**Fig. 2:**
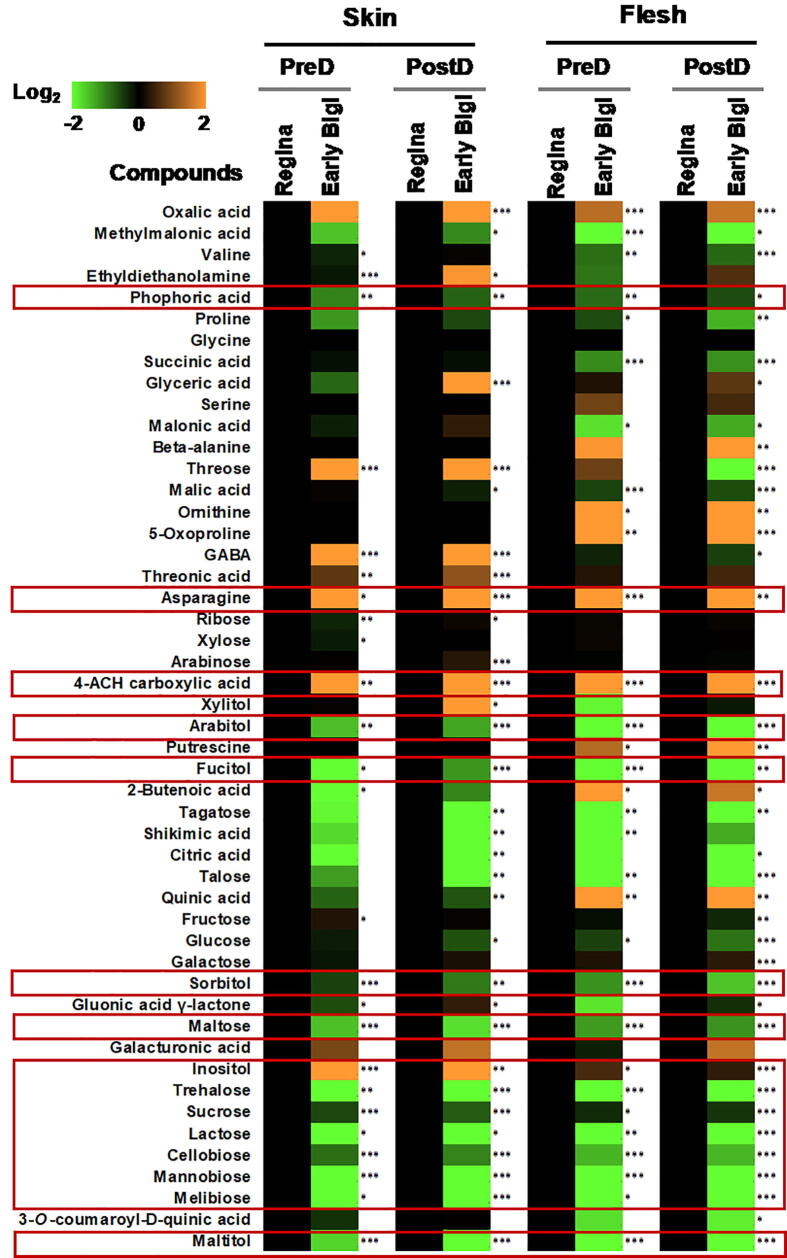
Influence of water treatment on fruit polar metabolome. Heatmaps indicate the difference in metabolite abundance between ‘Regina’ and ‘Early Bigi’ tissues samples (skin and flesh) at each time points (pre- and post-dipping). Additional experimental details as described in Fig. 1a. Each cell represents the log_2_FC and differences detected based on t-Student test; *P ≤ 0.05, **P ≤ 0.01 and ***P ≤ 0.001. Metabolites description and data provided in Supplementary Table 1.

The fact that fruit genotypes, ‘Early Bigi’ and ‘Regina’, differ in cracking (Fig. 1b and c) and watering behavior (Fig. 1d and e) suggests that there are differences in the adaption mechanisms controlling water-induced cracking. Therefore, we first investigated the possible changes in fruit primary metabolism between cultivars prior and just after water exposure. Using this approach, 49 primary polar metabolites were identified in the various samples (Supplementary Table 1). Principal component analysis (PCA) plot showed good separation among the analyzed samples (Supplementary Fig. 2a). From the total number of identified metabolites, the contents of 47 metabolites were changed in the comparison between cultivars (‘Regina’ *versus* ‘Early Bigi’), tissues (skin and flesh) and time point (pre- and post- dipping) (Fig. 2). For example, a comparison between cultivars indicated that 12 metabolites were decreased and 3 metabolites were increased in ‘Early Bigi’ cultivar in both tissues and time points. Particularly, various sugars (e.g. maltose, trehalose, sucrose, lactose, cellobiose, mannobiose and melibiose) and alcohols (e.g. arabitol, fucitol, sorbitol, maltitol) were reduced in ‘Early Bigi’ compared to ‘Regina’ (Fig. 2 & Supplementary Table 1). Moreover, the levels of asparagine, 4-ACH carboxylic acid and inositol were increased in ‘Early Bigi’, irrespectively of the tissue (skin and flesh) and time point (pre- and post- dipping) tested. Αn increase of threose, *γ*-Aminobutyric acid (GABA) and threonic acid in the skin of ‘Early Bigi’ was observed in both time points examined. In the flesh tissue of ‘Early Bigi’, a decrease of several acids (e.g. succinic acid, malonic acid, malic acid) as well as an increase of amino acids (e.g ornithine, 5-oxoproline), acids (e.g. 2-butenoic acid, quinic acid) were observed in both time points (Fig. 2 & Supplementary Table 1). Compared to ‘Regina’ samples, glyceric acid was increased in both tissues of ‘Early Bigi’ at post-dipping. Additionally, skin gluonic acid γ-lactone had lower abundance at pre-dipping and higher level at post-dipping in ‘Early Bigi’ than ‘Regina’ (Fig. 2 & Supplementary Table 1). Apart from genotypic differences, changes in metabolites were also observed when tissues (skin *versus* flesh) and time points (pre- *versus* post- dipping) were compared (Supplementary Fig. 2).

### 3.3 Transcriptomic analysis of sweet cherry genotypes, fruit tissues and water treatment

**Fig. 3:**
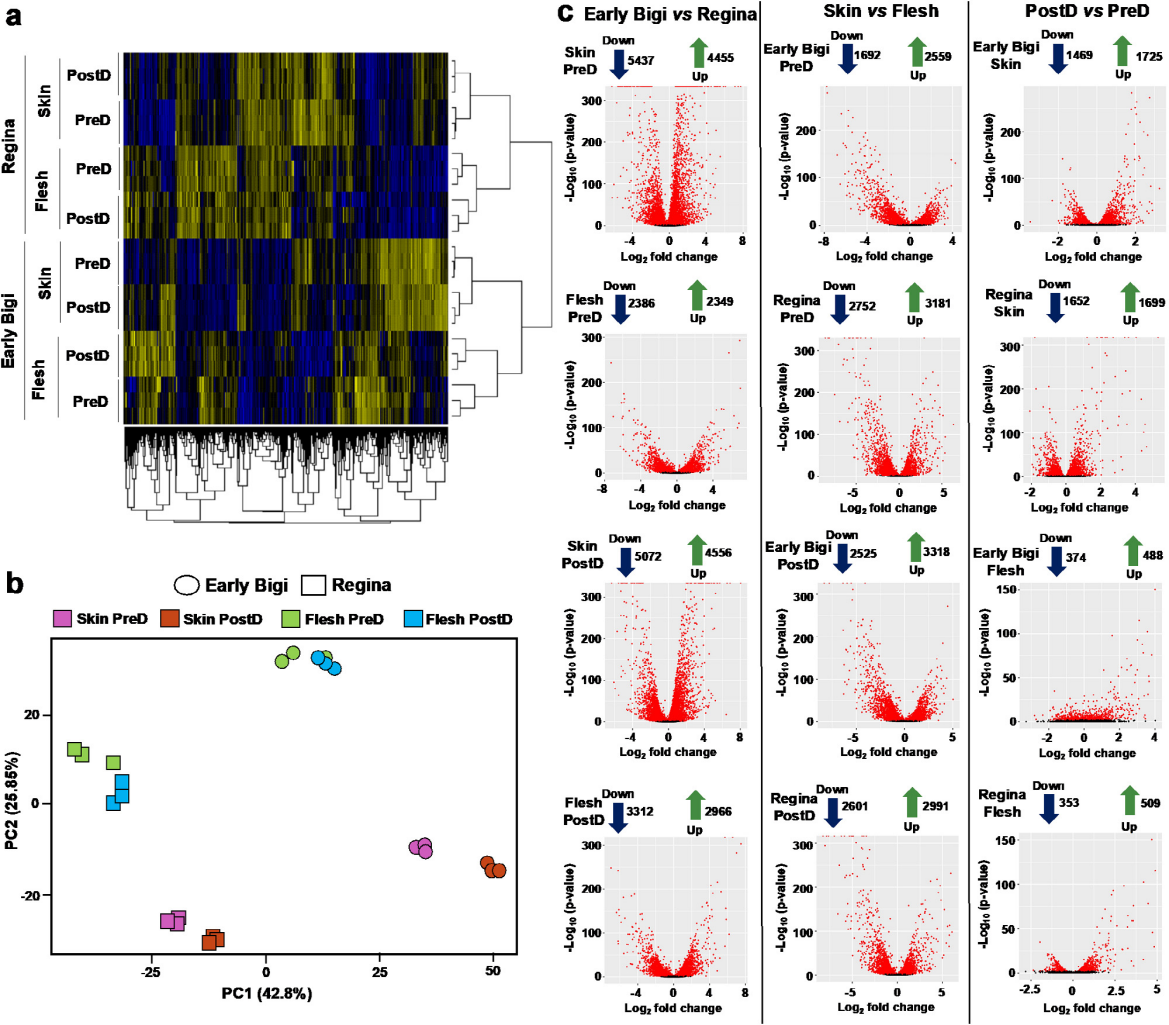
A global overview of the gene expression changes in sweet cherry tissues following water exposure. Additional experimental details as described in Fig. 1a. (a) Hierarchical clustering (Euclidean distance metric/spearman correlation method) (b) Principal Component Analysis (PCA) and (c) volcano plot depict clustering of differentially expressed (up-regulated and down-regulated) genes in ‘Early Bigi’ (*vs* ‘Regina’), skin (*vs* flesh) and post-dipping/PostD (*vs* pre-dipping/PreD) time.

**Fig. 4:**
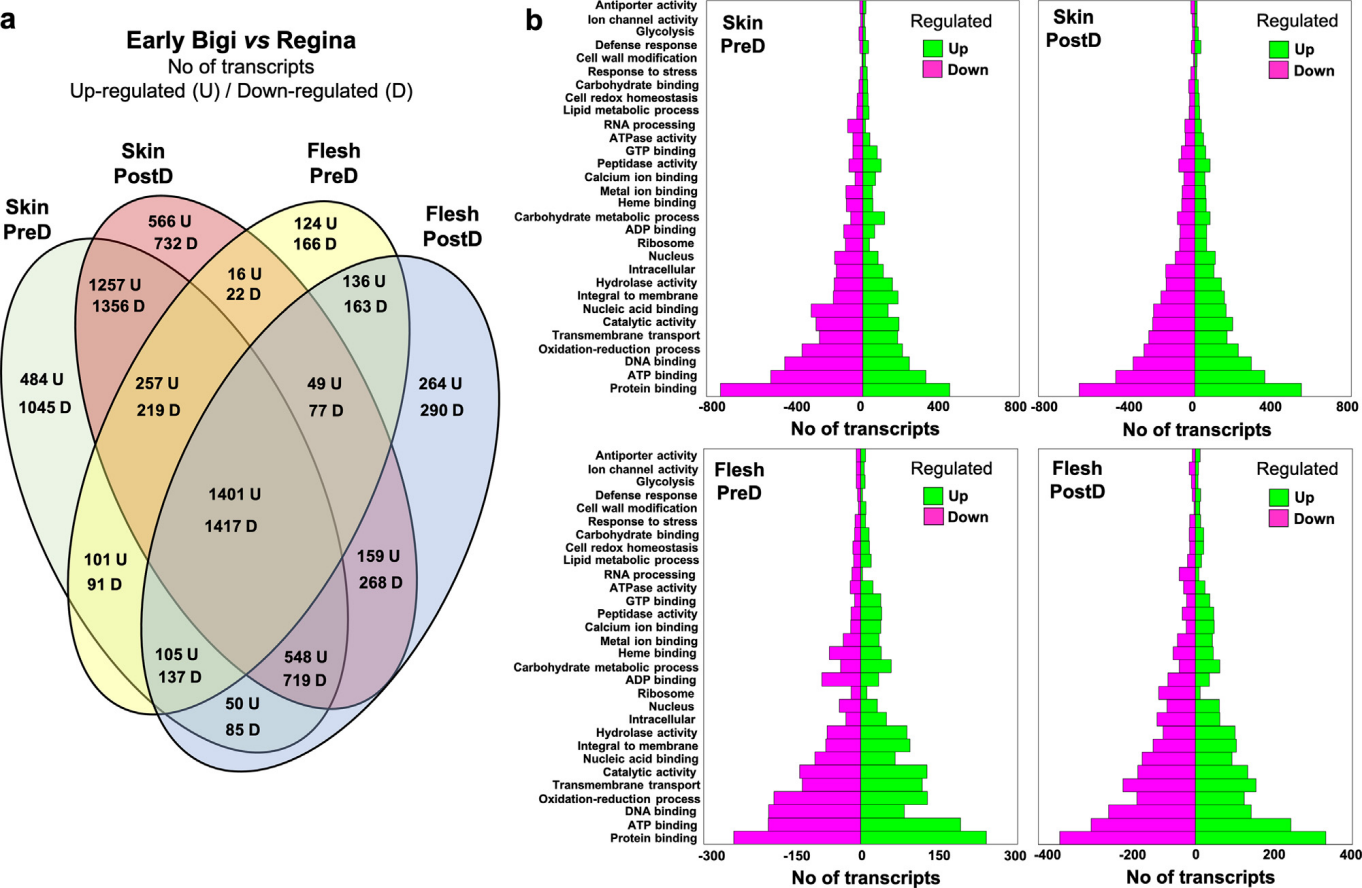
Changes in sweet cherry fruit transcriptome during water dipping and cracking development. (a) Venn diagram demonstrating the distribution of upregulated (U) or downregulated (D) genes in ‘Early Bigi’ skin and flesh tissues prior (PreD) and following dipping (PostD) compared to ‘Regina’ samples. (b) Enriched Gene Ontology (GO) categories in bar charts of ‘Early Bigi’ gene that upregulated (green) or downregulated (pink) across tissues and stages. The specific transcripts of GO categories are provided in the Supplementary Table 2. (For interpretation of the references to color in this figure legend, the reader is referred to the web version of this article.)

To further understand the different cracking response mechanisms between the two cultivars, transcriptomic analyses were conducted in flesh and skin tissues at pre- and post-dipping periods. Based on gene expression grouping through hierarchical clustering and PCA, a clear separation of the cultivars, fruit tissues (skin and flesh) and water immersion period (pre- and post-dipping in the skin tissue) were evidenced (Fig. 3a and b). The highest number of Differentially Expressed Genes (DEGs) were detected in the skin of ‘Early Bigi’ compared to ‘Regina’ in both pre- and post-dipping time while the lowest number of DEGs were found in post-dipping compared with pre-dipping period in the flesh of both cultivars (Fig. 3c–e; Supplementary Table 2). Interestingly a total of 1401 and 1417 genes were up- and down-regulated, respectively, regardless tissue and time point in ‘Early Bigi’ compared to ‘Regina’ cultivar (Fig. 4a) showing a genotype-dependent expression profile. Additionally, a high number of genes were found to demonstrate a tissue-specific expression profile with 2613 and 299 DEGs in ‘Early Bigi’ skin and flesh tissues, respectively, regardless the water-dipping treatment. At post-dipping time, 566, 264, and 159 genes were upregulated and 732, 290, and 268 genes were downregulated in the skin, flesh and in both tissues, respectively, of ‘Early Bigi’ compared to ‘Regina’ cultivar (Fig. 4a).

We further identified Gene Ontology (GO) categories that were over-represented in DEGs along with enrichment analysis in the various experimental combinations (cultivar/tissue/period) (Supplementary Table 2). Αn upregulation of genes related to cell wall modification was recorded in ‘Early Bigi’ tissues, suggesting that extensive changes in genes that encode cell wall enzymes were occurring in the cracking sensitive cultivar in response to water treatment (Fig. 4b). Following water dipping (PostD *vs* Pre-D), the number of genes related to ion channel activity and defense response were increased in both cultivars and tissues (Fig. 4b; Supplementary Table 2). The downregulation of several GTP binding genes in both tissues of ‘Early Bigi’ in response to dipping treatment (Fig. 4b; Supplementary Table 2), possibly indicates the effect of water exposure to their regulation.

### 3.4 Identification of differentially expressed genes putatively involved in water-induced fruit cracking

**Fig. 5:**
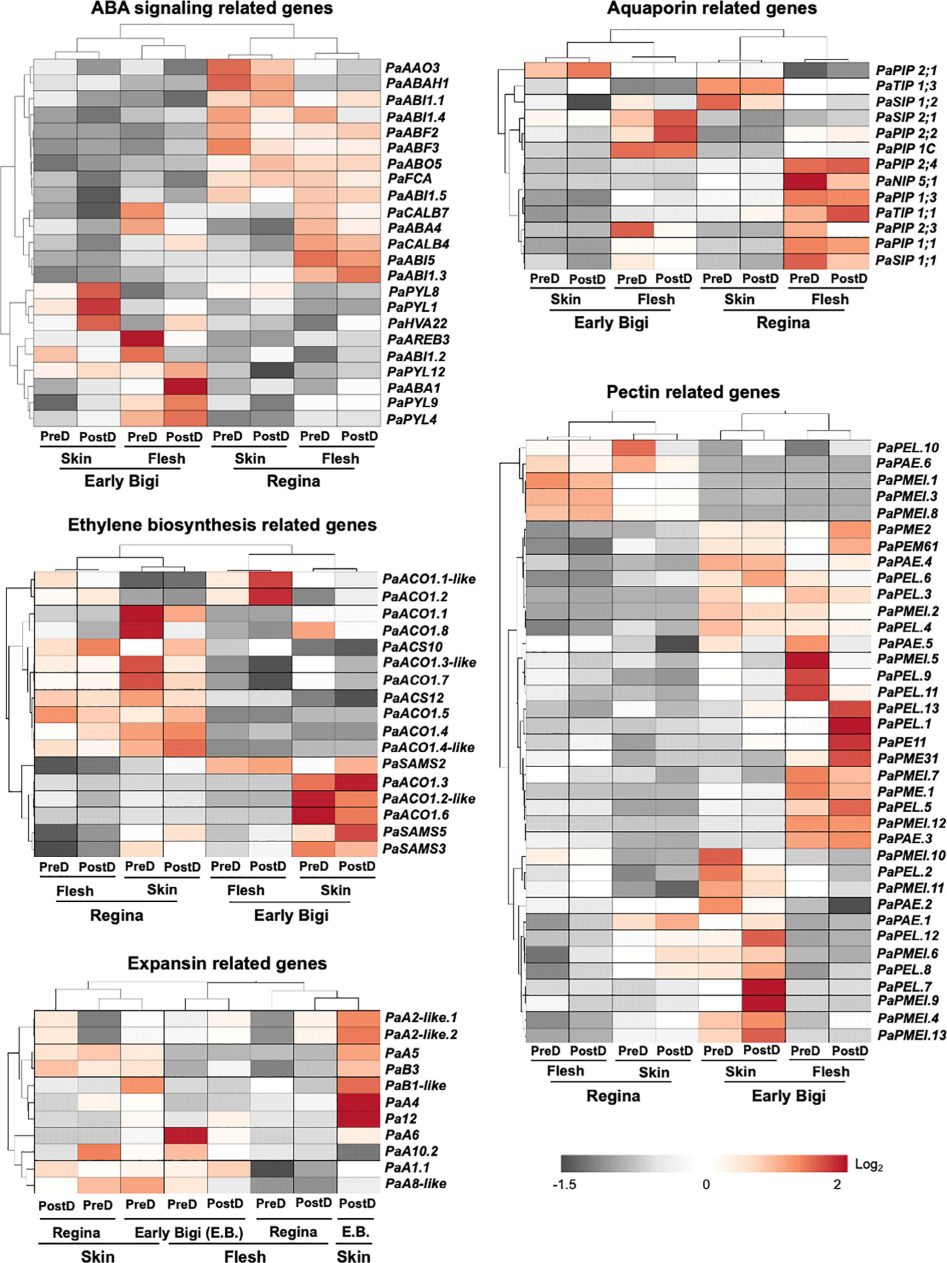
Specific categories of sweet cherry genes that affected by water exposure. Heat diagrams showing the temporal expression pattern in selected genes associated with ABA, ethylene, expansins, pectin and aquaporins signalling/metabolism/function in skin and flesh tissues of ‘Regina’ and ‘Early Bigi’ prior and after water dipping. Red cell indicates an increase and grey cell a decrease of gene expression. Additional experimental details as described in Fig. 1a. (For interpretation of the references to color in this figure legend, the reader is referred to the web version of this article.)

Based on previous studies [1], [17], [20], [21], [22], [23], [24], we focused on five gene categories, specifically ABA signaling, ethylene biosynthesis, expansins, pectin metabolism and aquaporins (Fig. 5, Supplementary Table 3), that could be involved in cracking development. The expression of genes related to ABA signaling, such as ABRE-binding factors (*PaABF2*, *PaABF3*), ABA overly‐sensitive 5 (*PaABO5*)*,* ABA insensitive (*PaABI*) and FLOWERING TIME CONTROL PROTEIN A (*PaFCA*) were downregulated in the cracking sensitive, ‘Early Bigi’ cultivar, while the ACTIN RESISTANCE 1 -LIKE (*PYL*) family genes were upregulated at post-dipping in both tissues of ‘Early Bigi’ (Fig. 5). Regarding ethylene biosynthesis, two distinct transcriptome profiles were identified. Some genes, including *S*-adenosylmethionines (*PaSAMS*) were upregulated only in ‘Early Bigi’ flesh while the other group of genes, including both 1-aminocyclopropane-1-carboxylic acid synthase (*PaACS*) and 1-aminocyclopropane-1-carboxylic acid oxidase (*PaACO*) were upregulated only in both tissues of ‘Regina’. It is also interesting that seven expansins-related genes were up-regulated in ‘Early Bigi’ skin after dipping; however, the four most abundant expansins in sweet cherry (*PaA1.1*, *PaA8-like*, *PaA10.2*) were upregulated in ‘Regina’ skin (Supplementary Table 3). Most of the members from aquaporin genes (7 out of 13) in flesh tissue, including *PaTIP 1;1*, *PaPIP 1;3*, *PaSIP 1;1* were de-activated after water treatment in ‘Early Bigi’, while the expression of *PaPIP 2;1*, was upregulated in ‘Early Bigi’ skin, specifically (Fig. 5 & Supplementary Table 3). The genes associated with pectin, including the most highly expressed genes in cherries (pectate lyase; *PaPEL.4*), showed the highest expression profiles in both skin and flesh of ‘Early Bigi’ (Supplementary Table 3).

The expression of several genes, including beta-fructofuranosidase (*PaINV*), alcohol dehydrogenase (*PaADH*), 1-acyl-*sn*-glycerol-3-phosphate acyltransferase (*PaPLSC*)*, PaPEL.7*, *PaSAMS5* that were identified as DEGs during the present RNA-seq experiments and were reported to be involved in cracking/water stress response, were validated by qRT-PCR (Supplementary Fig. 3).

### 3.5 Identification of single nucleotide variants in cracking related genes

**Fig. 6:**
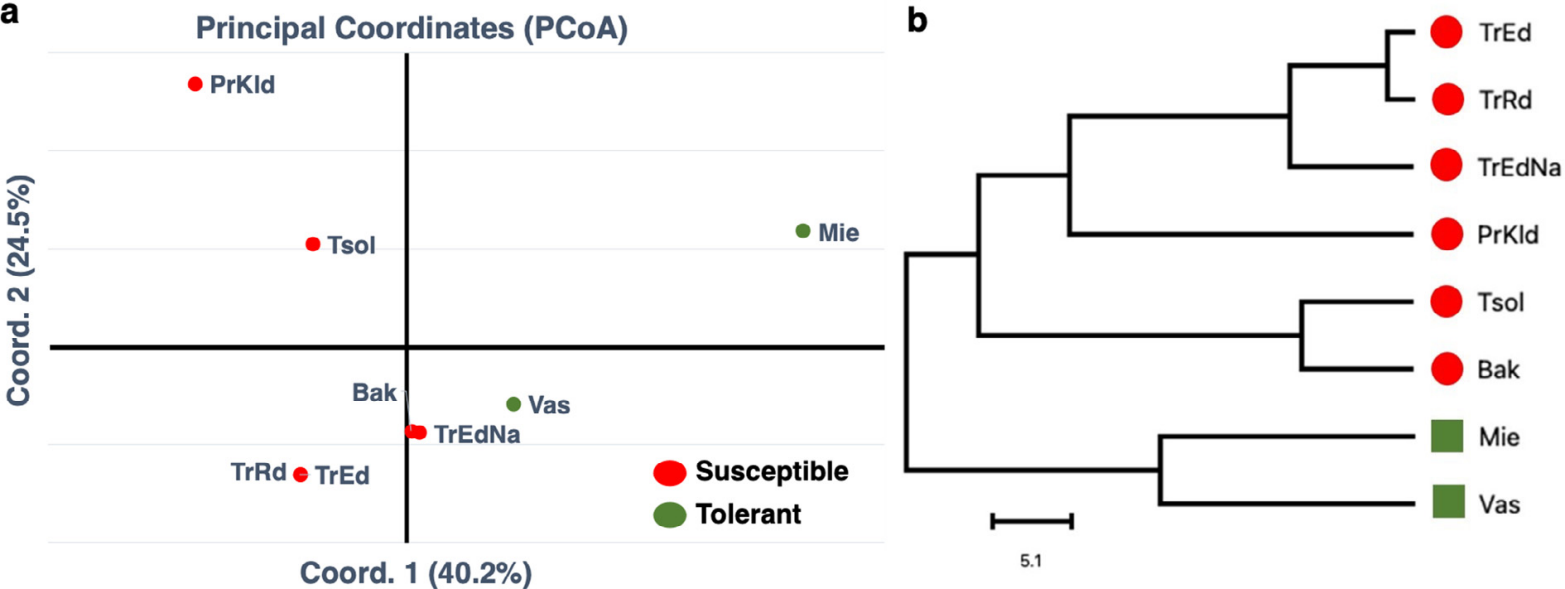
Identification and analysis of Single Nucleotide Variants (SNVs) located in the genomic sequence of pectin related hub-genes of eight sweet cherry cultivars. (a) Principal Coordinates Analysis (PCoA) and (b) Hierarchical clustering (Euclidean distance metric/spearman correlation method). Abbreviation of the cultivars: Bakirtzeika (Bak), Mieza (Mie), Proimo Kolindrou (PrKld), Tragana Edessis (TrEd), Tragana Edessis Naousis (TrEdNa), Tragana Rodochoriou (TrRd), Tsolakeika (Tsol), Vasileiadi (Vas). Red and green indicate cracking-susceptible and cracking-tolerant cultivars, respectively. For details, see the Supplementary Table 4. (For interpretation of the references to color in this figure legend, the reader is referred to the web version of this article.)

To obtain genotype-based insight into the regulatory events associated with water-induced cracking in sweet cherries at genome scale, we investigated the role and association of the above mentioned genes (Fig. 5) in cracking by using the whole genome re-sequencing data of eight sweet cherry cultivars from our recent work [38]. By employing cracking evaluation phenotypic data of these eight cultivars, two of them were classified as cracking relative tolerance while the rest as cracking susceptible (Supplementary Table 4). Subsequently, the single nucleotide variants (SNVs) of the coding regions of the pectin, aquaporin, expansin, ABA and ethylene related genes were studied to identify any association with the cracking phenotypes of these cultivars. Despite the identification of major SNVs into the other hub-genes, no clear clustering between cracking sensitive and tolerant cultivars was revealed regarding the aquaporin, expansin, ABA and ethylene related groups of genes (Supplementary Fig. 4 and Table 4), confirming the high complexity of the genotypic–phenotypic cracking relationships in sweet cherry [1], [2], [3], [4]. However, this genomic analysis revealed a strong association pattern of SNV of the pectin metabolism related genes with the selected cracking-phenotypes (Fig. 6, Supplementary Table 4), suggesting the possibility of using these genes as a universal marker of cracking in sweet cherry.

### 3.6 Water-responsive network in the tissues of the cracking-sensitive cultivar

**Fig. 7:**
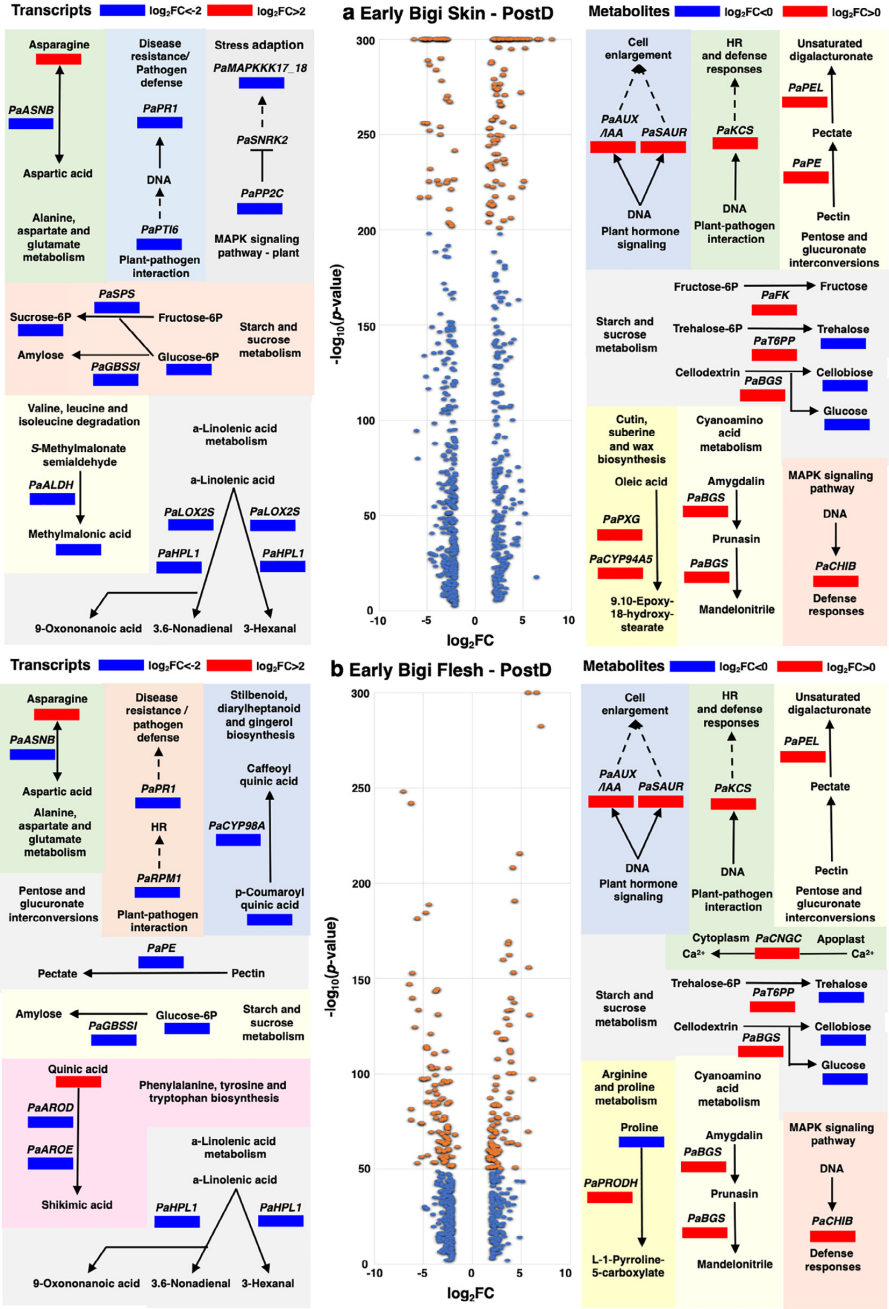
Gene, metabolites and networks that notably influenced by water dipping in both tissues of ‘Early Bigi’ cultivar. Strongly shifts of gene expressions (log_2_FC < |2| on volcano plots) and metabolites abundance (log_2_FC < or > 0) in ‘Early Bigi’ cultivar after post-dipping time point in (a) skin and (b) flesh tissues. Additional experimental detail as described in Fig. 1a. Metabolites and transcripts interactions were identified using the KEGG database and related pathways. Blue cell indicates a reduction (q- or p- value < 0.05) and red cell an increase (q- or p- value < 0.05) of tested variable. (For interpretation of the references to color in this figure legend, the reader is referred to the web version of this article.)

To understand the functions related to water in cracking symptoms we mapped in ‘Early Bigi’ tissues only the strongest post-dipping metabolic changes (log_2_FC < or > 0) together with the over-abundant (log_2_FC > |2|) DEGs in KEGG database (Fig. 7). This statistical-control approach revealed that 710 DEGs in skin (325 genes upregulated and 385 genes downregulated) and 557 DEGs in flesh (237 genes upregulated and 320 genes downregulated) were seriously affected by dipping (Fig. 7). Based on KEGG database, the major variation in metabolites level and gene expression was observed for eight pathways: the asparagine, starch and sucrose, a-linolenic acid, cyanoamino acid metabolism, pentose and glucuronate interconversions, plant hormone, MAPK signaling and plant-pathogen interaction (Fig. 7). Specifically, asparagine level increased and asparagine synthetase (*PaASNB*) gene that catalyzes asparagine biosynthesis was downregulated in both tissues of ‘Early Bigi’ following dipping. The levels of sugars glucose, trehalose and cellobiose (Fig. 2) along with granule-bound starch synthase (*PaGBSSI*) were decreased in both tissues of ‘Early Bigi’; however, trehalose 6-phosphate phosphatase (*PaT6PP*) and beta-glucosidase (*PaBGS*) that related to these sugars were increased (Fig. 7). Meanwhile, *PaBGS* is also participated in cyanoamino acid metabolism, as it metabolizes amygdalin into prunasin and then to mandelonitrile*.* The abundance of hydroperoxide lyase (*PaHPL1*), that participate in a-linolenic metabolism, were decreased in ‘Early Bigi’ skin and flesh (Fig. 7). Additionally, pectate degradation was activated, as evidenced by *PaPEL* up-regulation in both tissues of ‘Early Bigi’. Among genes known to be important for cell enlargement via plant hormone signaling, two sets of genes (auxin-responsive protein IAA; *PaAUX/IAA* and small auxin-up RNA; *PaSAUR*) was found to be induced in both tissues of ‘Early Bigi’ after dipping*.* Apart from that, the *PaKCS* and basic endochitinase B (*PaCHIB*) which involved in plant-pathogen interaction and in MAPK signaling, respectively, were uniquely up-regulated in ‘Early Bigi’ skin and flesh (Fig. 7). Genes, like peroxygenase (*PaPXG*) and fatty acid omega-hydroxylase (*PaCYP95A5*), whose products participate in cutin, suberin and wax biosynthesis were upregulated in ‘Early Bigi’ skin. Genes involved in pectin degradation, such as pectinesterase (*PaPE*), were differentially expressed in flesh (downregulated) and skin (upregulated) of ‘Early Bigi’ fruit exposed to dipping. In flesh of this cultivar, we found that a gene encoding cell-calcium influx (cyclic nucleotide gated channel; *PaCNGC*) was upregulated. Finally, the higher and lower levels of proline and quinic acid, respectively, could be associated with a reverse gene expression profile in ‘Early Bigi’ flesh (proline dehydrogenase; *PaPRODH*, 3-dehydroquinate dehydratase I; *PaAROD*, shikimate dehydrogenase; *PaAROE*; Fig. 7).

### 3.7 Genotype-specific pathways associated with water dipping and possibly with cracking expression signatures

**Fig. 8:**
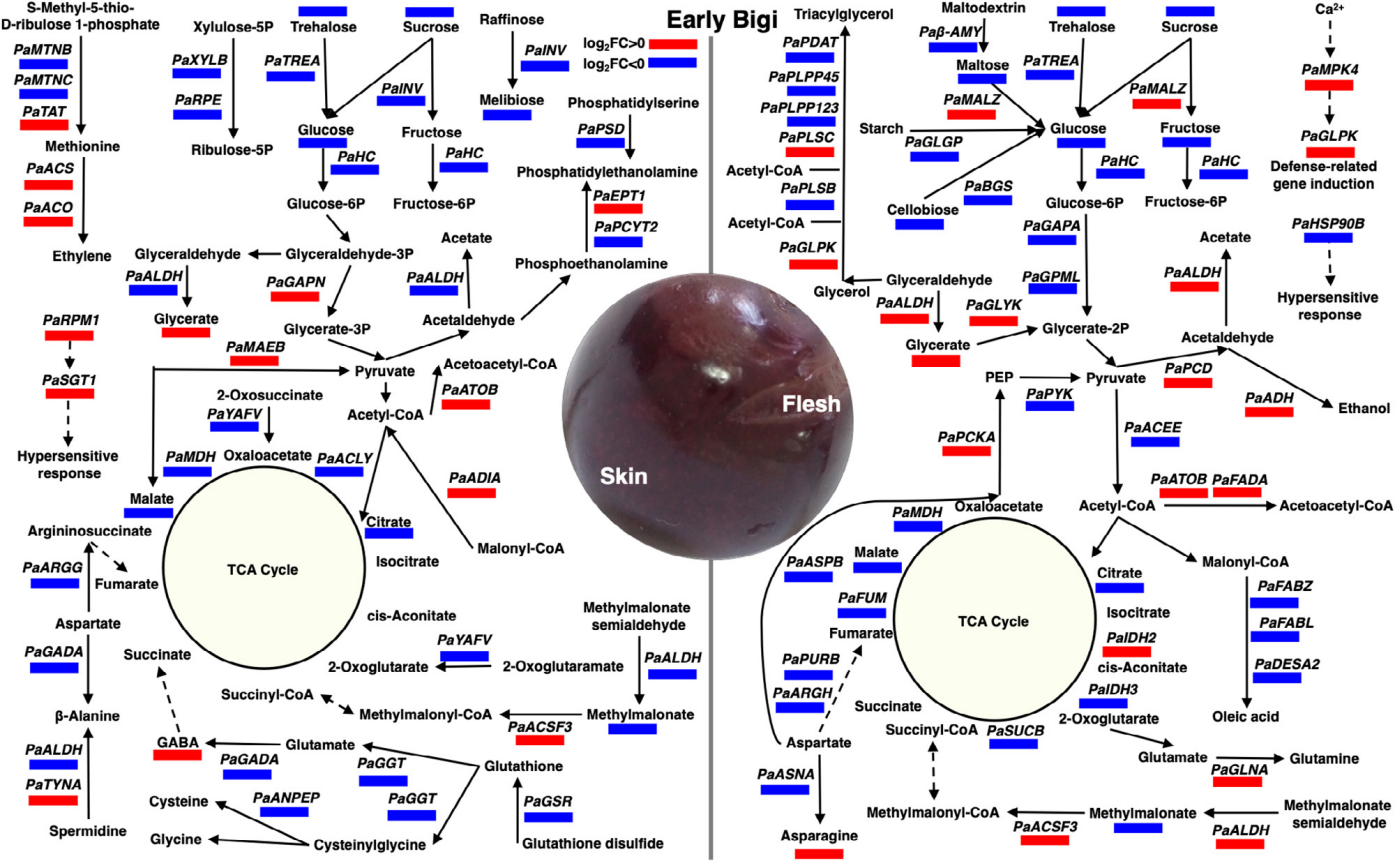
Global view of the tissue-specific pathways in ‘Early Bigi’ that associated with water exposure and possibly with cracking symptoms. Expression map of the water-derived metabolic changes in skin and flesh tissues of ‘Early Bigi’ cultivar compared with the corresponded tissues of ‘Regina’ at the post-dipping time point. Additional experimental details as described in Fig. 1a. Blue box indicates significant (q- or p- value < 0.05) decrease and red box implies significant (q- or p- value < 0.05) increase in the identified DEGs and metabolites. (For interpretation of the references to color in this figure legend, the reader is referred to the web version of this article.)

To obtain a more genotype-specific view of the effect of fruit dipping into cracking development, we compared all the significantly (*p- or q- value* ≤ 0.05) affected metabolites and genes in the tissues of both cultivar prior and after dipping. Subsequently, the obtained data were mapped to reference pathways in KEGG to identify the different networks that were de/activated in the skin and flesh of sweet cherry and their association with exposure to water and subsequently with cracking symptoms (Fig. 8). This analysis indicated that the two assessed tissues exhibit distinct and shared responses to water dipping, irrespectively of the cultivar. Some of the most striking common changes occurred in sugars metabolism and TCA cycle, which displayed a relative decrease in both tissues at post-dipping time, were depicted by the expression levels in alpha-trehalase (*PaTREA*), hexokinase (*PaHC*), malate dehydrogenase (*PaMDH*) genes and several metabolites such as sucrose, glucose, malate and citrate, whereas the gene, acetyl-CoA C-acetyltransferase (*PaATOB*), that is associated with acetyl-CoA metabolism, showed an upregulation in both tissues after dipping treatment (Fig. 8).

**Fig. 9:**
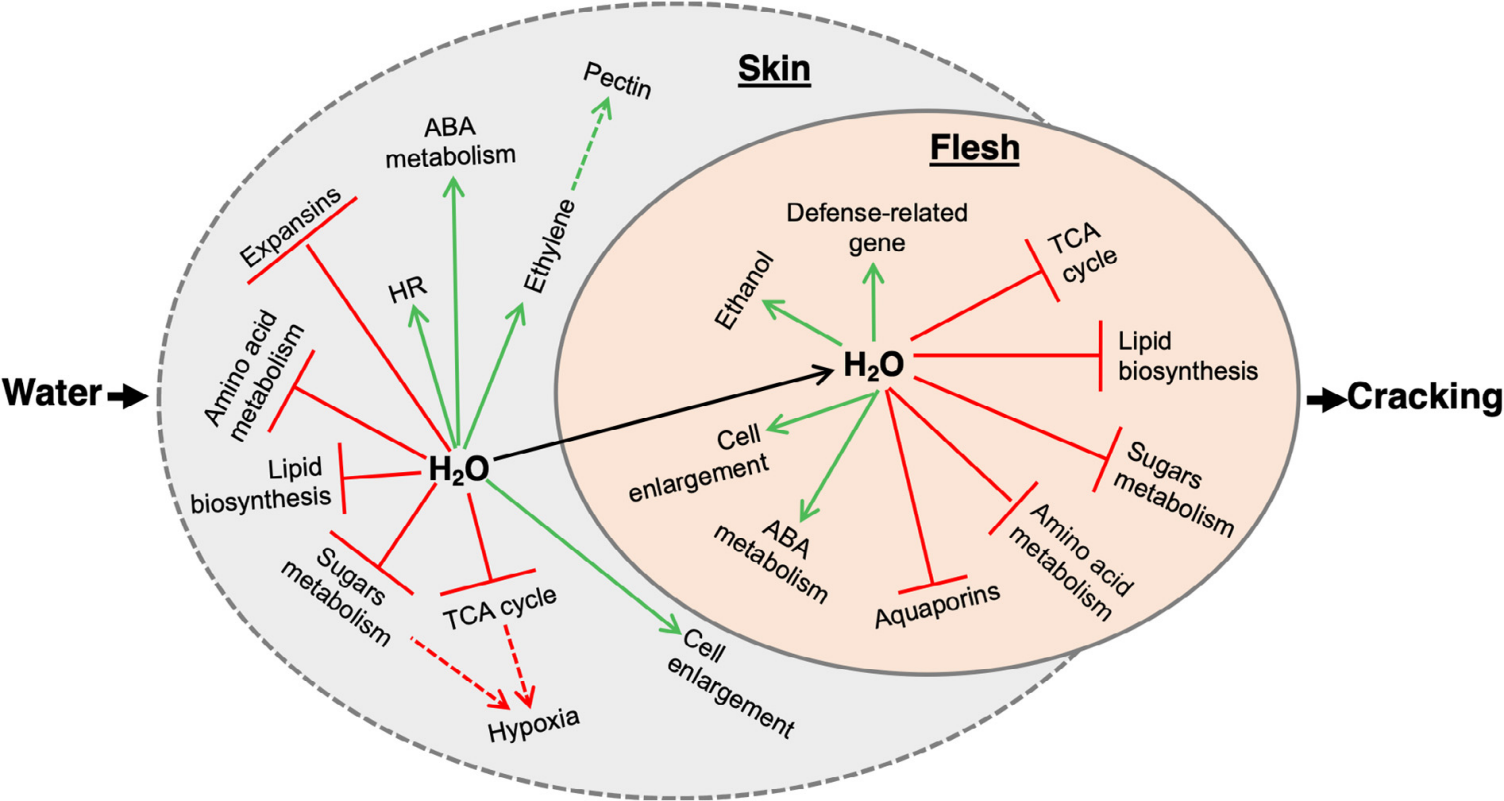
A putative genotype-specific model of water-induced key downstream metabolic events in skin and flesh 'Early Bigi’ tissues eventually leading fruit cracking.

Several distinct genotypic changes also appeared to be water affected either in skin or in flesh tissues of each tested cultivar. For example, the activation of pathways linked to ethylene production (tyrosine aminotransferase; *PaTAT*, *PaACS,* and *PaACO*) and to cell hypersensitive response (disease resistance protein; *PaRPM1* and small glutamine-rich tetratricopeptide repeat containing protein α; *PaSGT1*) was observed in ‘Early Bigi’ skin after dipping (Fig. 8). Meanwhile, the amino acid metabolism in ‘Early Bigi’ skin was depressed following incubation with water. Evidence for this response came from the observation that the expression of genes involved in glutathione metabolism (glutathione reductase; *PaGSR*, gamma-glutamyltranspeptidase/glutathione hydrolase; *PaGGT*, glutamate decarboxylase; *PaGADA,* and aminopeptidase N; *PaANPEP*) and in β-alanine metabolism (aldehyde dehydrogenase; *PaALDH*, and *PaGADA*) was reduced by water dipping (Fig. 8). In parallel, treatment with water resulted in the up-regulation of several genes associated with ethanol and acetate biosynthesis, like D-glycerate 3-kinase (*PaGLYK*), pyruvate decarboxylase (*PaPDC)*, *PaALDH,* and *PaADH* whereas water also caused the induction of defense-related genes (e.g mitogen-activated protein kinase 4; *PaMPK4* and glycerol kinase; *PaGLPK*) in the flesh of the sensitive cultivar ‘Early Bigi’ (Fig. 8). Finally, a large number of biosynthetic genes of triacylglycerol (glycerol-3-phosphate *O-*acyltransferase; *PaPLSB*, phosphatidate phosphatase; *PaPLPP123*, diacylglycerol diphosphate phosphatase; *PaPLPP45* and phospholipid:diacylglycerol acyltransferase; *PaPDAT*) and oleic acid (3-hydroxyacyl-[acyl-carrier-protein] dehydratase; *PaFABZ*, enoyl-[acyl-carrier protein] reductase I; *PaFABI* and acyl-[acyl-carrier-protein] desaturase; *PaDESA2*) were downregulated in the flesh tissue but without any significant change in the skin of ‘Early Bigi’ fruit subjected to water dipping (Fig. 8). The major skin and flesh responses that accompanied fruit cracking development in the susceptible ‘Early Bigi’ cultivar during water was illustrated in the Fig. 9. In both tissues of 'Early Bigi' cherries, genes along with metabolites related to cell enlargement and ABA signaling were increased; in contrast components of TCA cycle, sugar, and amino acids metabolism and lipid biosynthesis was decreased (Fig. 9). Moreover, genes related to expansins and aquaporins were downregulated in skin and flesh tissues, respectively. Finally, we found a strong upregulation of genes related to hypersensitive response (HR) and ethylene production in the skin samples as well as in genes involved in cell defense and ethanol in the flesh tissue (Fig. 9).

## 4 Discussion

Physiological data indicated that water dipping induced cracking damage in ‘Early Bigi’ fruit, as shown by the higher cracking index (Fig. 1b), cracking classes (Fig. 1c), EC-release on the water (Fig. 1d), and water absorption (Fig. 1e), thus confirming the susceptibility of this cultivar to cracking [8]. The significantly different response of ‘Regina’ in these cracking traits (Fig. 1b, c, d & e) is consistent with previous reports showing that this is a relative cracking tolerant cultivar [4], [8], [43], [44]. Therefore, the aim of this work was to determine the extent to which tissue-specific metabolic and transcript profiling driven by water might, in part, explain the co-differential cracking phenotypes in these genotypes.

The present work identified a large number of sweet cherry genes that are differentially expressed between ‘Regina’ and ‘Early Bigi’ tissues. Particularly, we found that several abscisic acid (ABA) related genes, such ABA-induced protein (*PaHVA22*) and ABA‐deficient (*PaABA1*) were induced in the tissues of ‘Early Bigi’ following water dipping (Fig. 5), indicating that water uptake may influence ABA signaling. It has been shown that ABA application enhanced water movement into tomato fruit and increased fruit cracking [20], which lent support to the current data. It has also been established that ABA stimulates ethylene production in sweet cherries, thus pointing that cherry fruits may have the potential to synthesize ethylene in an ABA-dependent manner [45]. Thus, the up-regulation of various ABA receptors, such as *PaPYL*, *PaHVA22*, and *PaABA1* in both tissues of ‘Early Bigi’ following dipping could be coupled to the increased expression levels of key ethylene biosynthesis genes *PaSAMS*s and *PaACO1*s (Fig. 5). Taken together, these data suggest the occurrence of a biological connection between ABA and ethylene signaling during water-induced cracking in sweet cherry.

The results presented herein indicated that most of the aquaporin genes, including *PaTIP 1;1*, *PaPIP 1;3*, *PaSIP 1;1* were activated by water in ‘Regina’ flesh (Fig. 5), highlighting the possible role of these genes in water transport across plasma membranes. The expressions patterns of these genes, however, did not parallel to the lower cracking symptoms (Fig. 5) that are usually associated with reduced water efflux [24], [46]. A possible explanation may be the fact that the skin of sweet cherry is markedly strained [47]. It has been proposed that the tangential strain in the skin (unmatched by epidermal cell division) causes an increase in an epidermal cell’s area (periclinal) but a decrease in its thickness (anticlinal) [46]. Consequently, the epidermal cell surface area increases, while its volume decreases. A decrease in resistance of the plasma membrane to water transport due to an opening of aquaporin would facilitate an easier water partition between symplast and apoplast, thereby alleviating both stress and strain. Based on the above hypothesis, this might decrease the likelihood of sweet cherry cracking [46]. An alternative suggestion for the observed activation of aquaporins in ‘Regina’ flesh (Fig. 5) could be that cracking in sweet cherry is a localized phenomenon that is not related to the net fruit water balance (“turgor hypothesis”) but is mainly the consequence of more local exposure of the fruit skin to liquid-phase water (“zipper hypothesis”) [43].

The results of the present study further suggest that the cracking behavior of each cultivar could be related to genotype-dependent differences in cell wall physiochemical properties, particularly evidenced after a watering episode. For example, the up-regulation of several expansin genes, including *PaA4*, *Pa12 and PaB1-like* in ‘Early Bigi’ skin after dipping could implied a water-induced faster skin expansion phase in this cultivar, leading to cracking development. At the same time, high expression levels of genes associated with pectin, such as *PaPEL.3, 4* and *6* in wetted ‘Early Bigi’ samples (Fig. 5) is likely to reflect enhanced cuticle deposition to repair the extensive cracks and/or strengthen a weaker structure, as proposed in tomato [48]. In support to the role of pectin metabolism in sweet cherry fruit cracking, our genomic data based on single nucleotide variants disclosed that pectin metabolism related genes were closely linked to cracking index in eight tested cultivars (Fig. 6), suggesting that pectin globally plays an important role in sweet cherry cracking outcome irrespective of genotype, probably to control cell wall swelling.

The analysis of strongly shifts (log_2_FC < |2|) of transcript expression and metabolites abundance after dipping (Fig. 7), revealed tissue-specific processes in ‘Early Bigi’, possibly leading to cracking defects (Fig. 1b & c). This result indicates that water-induced cracking ‘Early Bigi’ is associated with the up-regulation of cell enlargement genes such as *PaAUX/IAA* and *PaSAUR* in both tissues examined (Fig. 7). These genes are mainly expressed during fruit growth [49], [50], denoting that the water-intake into the sweet cherry fruit increases cell wall pressure and subsequently activates genes related to both skin and flesh cell enlargement. Current data further uncovered that several defense-related genes were affected by water dipping in ‘Early Bigi’. For instance, we found an up-regulation of *PaKCS* (fatty acid elongases) and *PaCHIB* (bacterial chitinases), whose role in plant defense is well documented [51], [52]; such behavior might represent an adaptive strategy against pathogens in ‘Early Bigi’ following excessive water absorption (Fig. 1e) and cracking symptoms (Fig. 1b and c).

Of particular interest is the observation that the activation of beta-glucosidase (*PaBGS*) was accompanied with low abundance of glucose (Fig. 2) in both tissues of ‘Early Bigi’ at post dipping stage (Fig. 7). Given that *PaBGS* is linked to (i) conversion of amygdalin into prunasin [53], and (ii) cell wall component cellodextrin degradation into cellobiose and glucose [54], we hypothesize that the observed induction of *PaBGS* can account for the reduced glucose level and is likely to involve a complex interplay among sucrose homeostasis (e.g., cellobiose, glucose), cyanoamino acid metabolism (e.g., amygdalin, prunasin) and water-driven cracking networks. We also showed that the exposure of ‘Early Bigi’ tissues to water induced asparagine accumulation but also downregulated the glutamine-dependent asparagine synthetase (*PaASNB*) (Fig. 7) that converts asparagine into aspartic acid. The link between asparagine and cracking responses is by no means unprecedented, given that a strong positive correlation was detected between asparagine and cracking index classification among several sweet cherry cultivars [8].

A cluster of genes involved in sugars and TCA cycle metabolism was found to strongly affected (*p- or q- value* ≤ 0.05) between pre- and post-dipping time while also being differentially expressed in ‘Regina’ and ‘Early Bigi’ fruit (Fig. 8). The lower accumulation of several soluble sugars, especially sucrose and glucose that observed in ‘Early Bigi’ (Fig. 2) could play a less-effective osmoregulatory role and would increase the fruit permeability, thereby would allow more water entry in this cultivar (Fig. 8) when exposed to water conditions [8], such as dipping-induced cracking (Fig. 1b and c) [55]. Sugars metabolism could be also associated with TCA cycle because of the main source of acetyl-CoA, which supplies the TCA cycle, they are coming from sugars metabolism through pyruvate [56]. Labelling studies suggest that carboxylic acid metabolism via TCA cycle with non-cyclic flux modes regulate the metabolic demands of the cells under stress, like hypoxia [55], [57]. Oxygen depletion is known to occur in water stress conditions [58] and the cyclic flux mode in TCA cycle under aerobic conditions became less evident under hypoxia [55]. In support to putative role of a transient hypoxia phenomenon in cracking, our data showed that a large group of TCA- associated genes (e.g. *PaALDH, PaGLYK, PaACLY, PaMDH*) and metabolites (e.g. sucrose, glucose, malate, citrate) undergo dramatic shifts in their accumulation following water treatment. In addition, the level of glyceric acid was increased in both ‘Early Bigi’ tissues following water dipping (Fig. 2), probably implicates an interconnection of this treatment to low oxygen since glycerate is related to hypoxia in plants [59]. It is therefore possible that water uptake in intercellular air space of sweet cherry fruit skin and flesh induced hypoxia conditions, as proposed in mangosteen fruit [60]. Meanwhile, several genes related to ethanol accumulation (i.e., *PaPCD* and *PaADH*) were induced by dipping in ‘Early Bigi’ tissues (Fig. 8), demonstrating that cracking sensitivity is likely to be dependent on additional uncharacterized pathways.

Another notable feature of the present analysis was the downregulation of genes associated with lipid biosynthesis (i.e., *PaHPL* and lipoxygenase; *PaLOX2S*) in ‘Early Bigi’ skin due to water dipping, suggesting an inhibitory role of forcing water supply in lipid biosynthesis. Consistent with our results, it has been recently reported that the expression of several genes involved in lipid biosynthesis was reduced in cracked jujube fruits [61]. Analogously, we observed lower expression of genes associated with lipid biosynthesis, such as *PaPLSB, PaPLPP123, PaPLPP45, PaPDAT* in ‘Early Bigi’ flesh samples. It is well documented that the lipid biosynthesis in the skin tissue offers protection against stresfull conditions, but in the flesh helps to maintain membrane lipid homeostasis and integrity [62], [63]. Hence, the downregulation of lipid biosynthesis-related genes could be associated with flesh membrane dysfunctions, possibly reflecting the inability of ‘Early Bigi’ fruit to cope with massive water exposure.

## 5 Conclusion

In this report, we generated for the first-time extensive transcriptome data of two sweet cherry cultivars that exhibited contrasting cracking physiognomy in response to water exposure. By integrating and comparing the metabolic responses triggered by water in these cultivars, we propose a genotype-specific model on how the water exposure induced major metabolic output in the skin and flesh tissues of the cracking susceptible ‘Early Bigi’ cultivar (Fig. 8). Water mainly disturbs core metabolism, particularly genes related to TCA cycle, sugars, and amino acid metabolism but we also highlighted that genes related to expansins in the skin as well as aquaporins and lipids in the flesh could be linked to ‘Early Bigi’ cracking. Thanks to the availability of genomic and cracking data of various cultivars, our single nucleotide variants analysis provide evidence that pectin metabolism related genes may be globally involved in sweet cherry fruit response to cracking. The expression of several genes in this cultivar that involved in ABA and ethylene signaling, defense responses and ethanol biosynthesis were induced by water in a tissue-specific manner. Overall, we identified various water-affected individual components and metabolic processes, mostly unreported in previous studies, which provides insight into sweet cherry cracking. Given that the breeding for cracking tolerance has always been considered a key priority in sweet cherry research, our study offers an important background for the identification of key genes involved in this phenomenon, thereby provide plant breeders with better screening criteria for cracking-tolerant cherry cultivars.

## Funding

This research is co-financed by Greece and the European Union (European Social Fund- ESF) through the Operational Programme ‘Support for Researchers with Emphasis on Young Researchers’ in the context of the project ‘EDBM103-Part B, 2020-2022′ (MIS: 5047887).

## CRediT authorship contribution statement

**Michail Michailidis:** Conceptualization, Methodology, Investigation, Data curation, Formal analysis, Visualization, Writing – original draft. **Evangelos Karagiannis:** Investigation, Formal analysis. **Christos Bazakos:** Formal analysis, Data curation, Visualization. **Georgia Tanou:** Formal analysis, Writing – review & editing. **Ioannis Ganopoulos:** Conceptualization, Writing – review & editing. **Athanassios Molassiotis:** Conceptualization, Validation, Project administration, Supervision, Writing – review & editing.

## Declaration of Competing Interest

The authors declare that they have no known competing financial interests or personal relationships that could have appeared to influence the work reported in this paper.
