# Supplementary material for: Genotype- and tissue-specific metabolic networks and hub genes involved in water-induced distinct sweet cherry fruit cracking phenotypes
Source: Comput Struct Biotechnol J. 2021 Sep 28;19:5406–20. doi: 10.1016/j.csbj.2021.09.030 (PMC8501671; doi:10.1016/j.csbj.2021.09.030)
Supplement: Supplementary data 1 [file mmc1.pptx]

## Slide 1
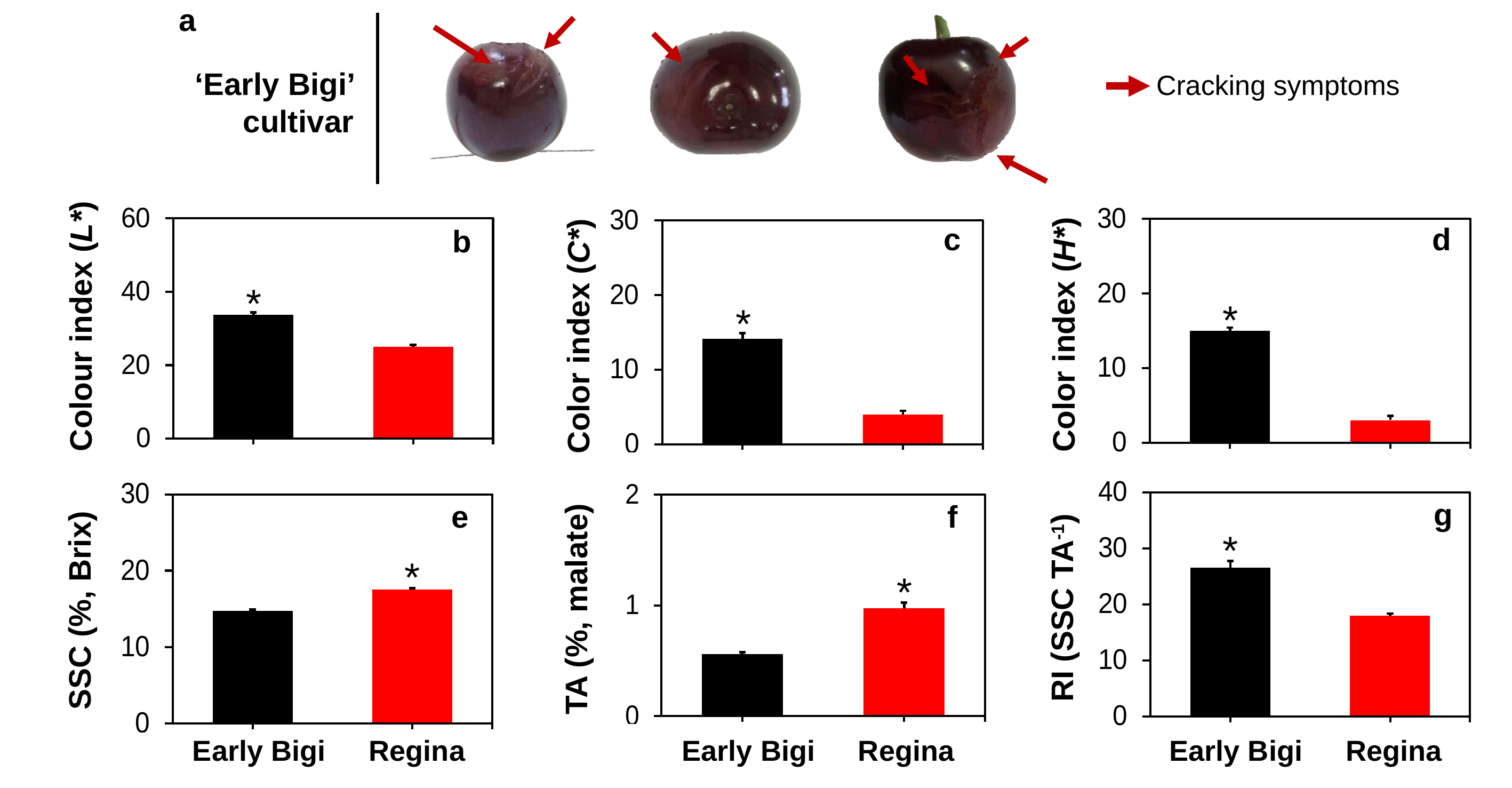

a
‘Early Bigi’ cultivar
Cracking symptoms
d
c
b
*
*
*
Colour index (L*)
Color index (H*)
Color index (C*)
g
e
f
*
*
*
RI (SSC TA-1)
TA (%, malate)
SSC (%, Brix)
Early Bigi
Regina
Early Bigi
Regina
Early Bigi
Regina
