# Supplementary material for: Genotype- and tissue-specific metabolic networks and hub genes involved in water-induced distinct sweet cherry fruit cracking phenotypes
Source: Comput Struct Biotechnol J. 2021 Sep 28;19:5406–20. doi: 10.1016/j.csbj.2021.09.030 (PMC8501671; doi:10.1016/j.csbj.2021.09.030)
Supplement: Supplementary data 2 [file mmc2.pptx]

## Slide 1
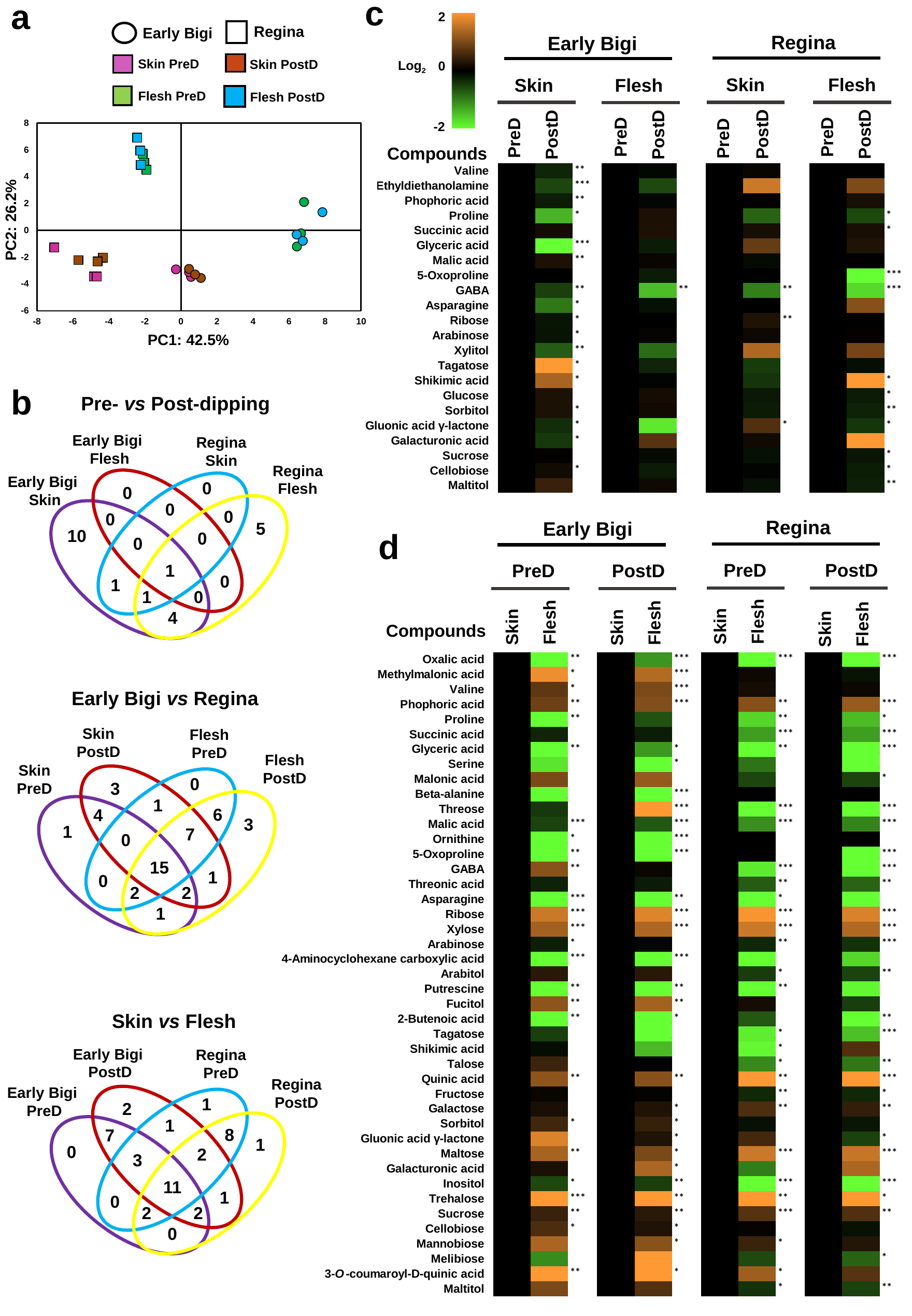

c
a
2
Regina
Early Bigi
Regina
Early Bigi
Skin PreD
Skin PostD
Log2
0
Skin
Flesh
Skin
Flesh
Flesh PreD
Flesh PostD
-2
PostD
PostD
PostD
PostD
PreD
PreD
PreD
PreD
Compounds
PC2: 26.2%
PC1: 42.5%
b
Pre- vs Post-dipping
Early Bigi
Flesh
Regina
Skin
Regina
Flesh
Early Bigi
Skin
0
0
0
0
0
Regina
Early Bigi
5
10
d
0
0
PreD
PostD
PreD
PostD
1
0
1
1
0
4
Flesh
Flesh
Flesh
Flesh
Skin
Skin
Skin
Skin
Compounds
Early Bigi vs Regina
Skin
PostD
Flesh
PreD
Flesh
PostD
Skin
PreD
0
3
1
6
4
3
1
7
0
15
1
0
2
2
1
Skin vs Flesh
Early Bigi
PostD
Regina
PreD
Regina
PostD
Early Bigi
PreD
1
2
1
8
7
1
0
2
3
11
1
0
2
2
0
