# Supplementary material for: Genotype- and tissue-specific metabolic networks and hub genes involved in water-induced distinct sweet cherry fruit cracking phenotypes
Source: Comput Struct Biotechnol J. 2021 Sep 28;19:5406–20. doi: 10.1016/j.csbj.2021.09.030 (PMC8501671; doi:10.1016/j.csbj.2021.09.030)
Supplement: Supplementary data 3 [file mmc3.pptx]

## Slide 1
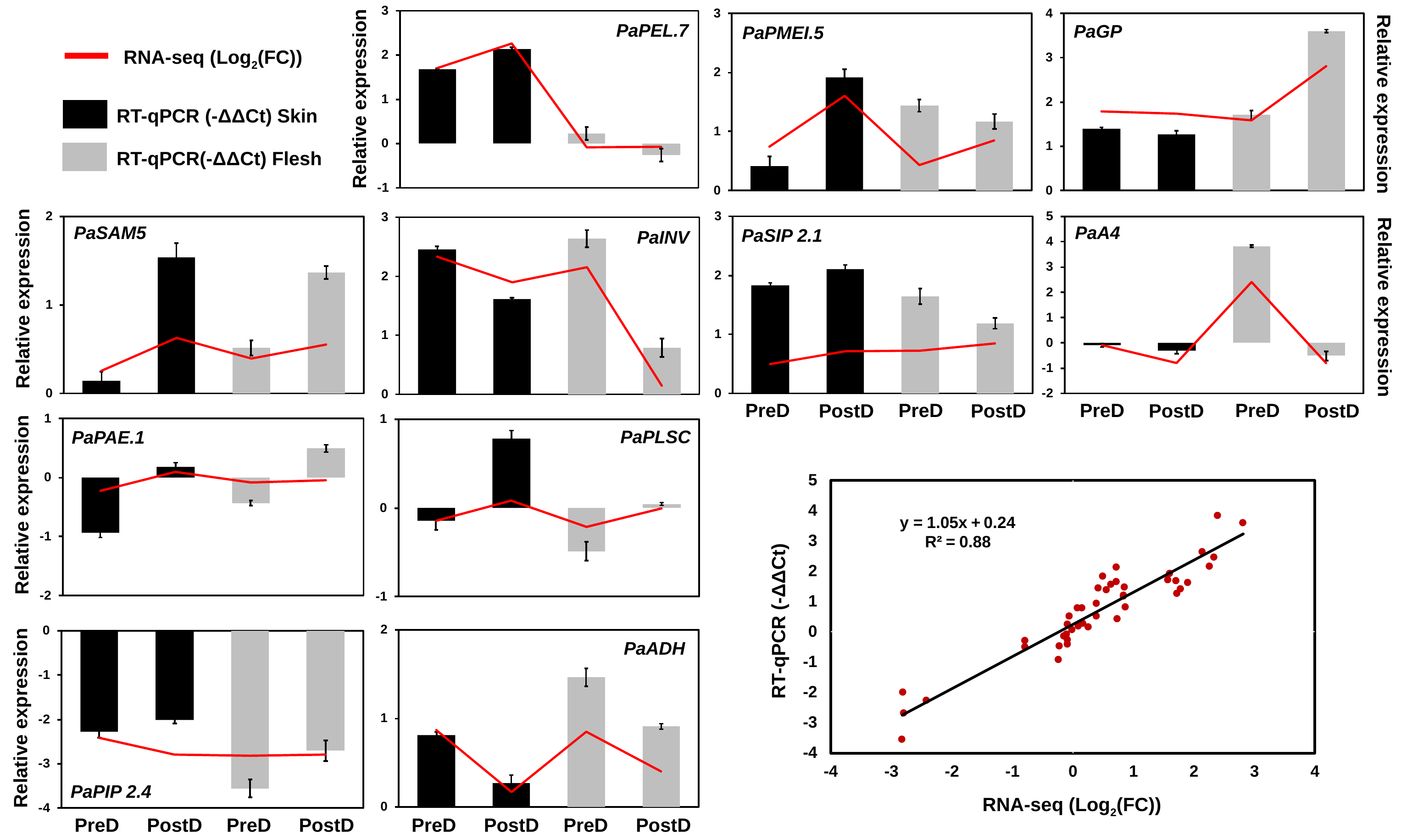

PaPEL.7
PaGP
PaPMEI.5
RNA-seq (Log2(FC))
Relative expression
Relative expression
RT-qPCR (-ΔΔCt) Skin
RT-qPCR(-ΔΔCt) Flesh
PaA4
PaSAM5
PaSIP 2.1
PaINV
Relative expression
Relative expression
PreD
PreD
PreD
PreD
PostD
PostD
PostD
PostD
PaPLSC
PaPAE.1
Relative expression
RT-qPCR (-ΔΔCt)
PaADH
Relative expression
PaPIP 2.4
RNA-seq (Log2(FC))
PreD
PreD
PreD
PreD
PostD
PostD
PostD
PostD
