# Supplementary material for: Genotype- and tissue-specific metabolic networks and hub genes involved in water-induced distinct sweet cherry fruit cracking phenotypes
Source: Comput Struct Biotechnol J. 2021 Sep 28;19:5406–20. doi: 10.1016/j.csbj.2021.09.030 (PMC8501671; doi:10.1016/j.csbj.2021.09.030)
Supplement: Supplementary data 4 [file mmc4.pptx]

## Slide 1
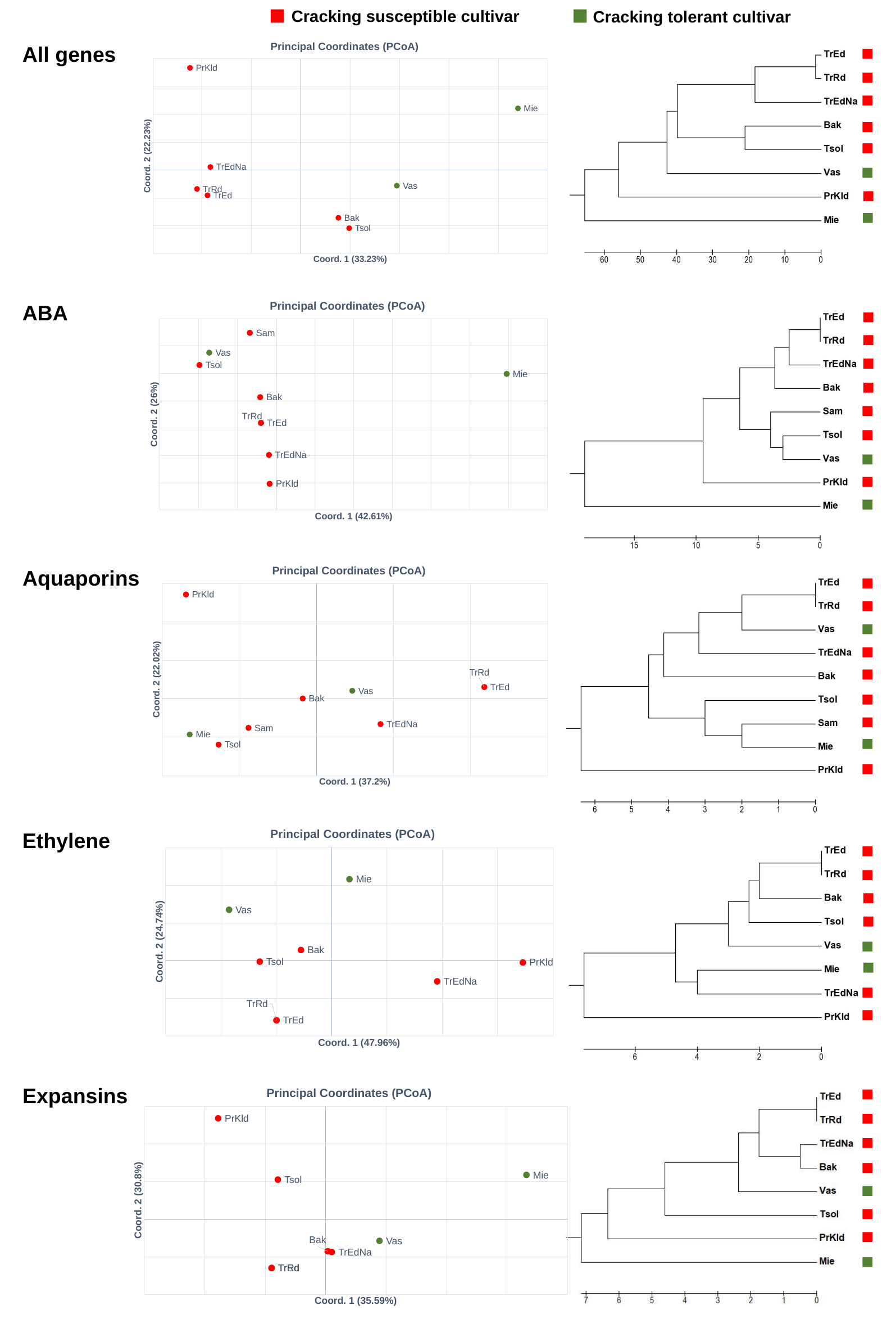

Cracking susceptible cultivar
Cracking tolerant cultivar
All genes
ABA
Aquaporins
Ethylene
Expansins
